# Thiazolidinediones lower the risk of pneumonia in patients with type 2 diabetes

**DOI:** 10.3389/fmicb.2023.1118000

**Published:** 2023-02-17

**Authors:** Fu-Shun Yen, James Cheng-Chung Wei, Yu-Tung Hung, Chung Y. Hsu, Chii-Min Hwu, Chih-Cheng Hsu

**Affiliations:** ^1^Dr. Yen’s Clinic, Taoyuan, Taiwan; ^2^Institute of Medicine, Chung Shan Medical University, Taichung, Taiwan; ^3^Department of Medicine, Chung Shan Medical University Hospital, Taichung, Taiwan; ^4^Graduate Institute of Integrated Medicine, China Medical University, Taichung, Taiwan; ^5^Management Office for Health Data, China Medical University Hospital, Taichung, Taiwan; ^6^College of Medicine, China Medical University, Taichung, Taiwan; ^7^Graduate Institute of Biomedical Sciences, China Medical University, Taichung, Taiwan; ^8^Faculty of Medicine, National Yang-Ming Chiao Tung University School of Medicine, Taipei, Taiwan; ^9^Section of Endocrinology and Metabolism, Department of Medicine, Taipei Veterans General Hospital, Taipei, Taiwan; ^10^Institute of Population Health Sciences, National Health Research Institutes, Miaoli, Taiwan; ^11^Department of Health Services Administration, China Medical University, Taichung, Taiwan; ^12^Department of Family Medicine, Min-Sheng General Hospital, Taoyuan, Taiwan; ^13^National Center for Geriatrics and Welfare Research, National Health Research Institutes, Miaoli, Taiwan

**Keywords:** all-cause pneumonia, bacterial pneumonia, death, invasive mechanical ventilation, pioglitazone

## Abstract

**Introduction:**

We conducted this study to compare the risk of pneumonia between thiazolidinedione (TZD) use and nonuse in persons with type 2 diabetes (T2D).

**Methods:**

We identified 46,763 propensity-score matched TZD users and nonusers from Taiwan’s National Health Insurance Research Database between January 1, 2000, and December 31, 2017. The Cox proportional hazards models were used for comparing the risk of morbidity and mortality associated with pneumonias.

**Results:**

Compared with the nonuse of TZDs, the adjusted hazard ratios (95% CI) for TZD use in hospitalization for all-cause pneumonia, bacterial pneumonia, invasive mechanical ventilation, and death due to pneumonia were 0.92 (0.88–0.95), 0.95 (0.91–0.99), 0.80 (0.77–0.83), and 0.73 (0.64–0.82), respectively. The subgroup analysis revealed that pioglitazone, not rosiglitazone, was associated with a significantly lower risk of hospitalization for all-cause pneumonia [0.85 (0.82–0.89)]. Longer cumulative duration and higher cumulative dose of pioglitazone were associated with further lower adjusted hazard ratios in these outcomes compared to no-use of TZDs.

**Discussion:**

This cohort study demonstrated that TZD use was associated with significantly lower risks of hospitalization for pneumonia, invasive mechanical ventilation, and death due to pneumonia in patients with T2D. Higher cumulative duration and dose of pioglitazone were associated with a further lower risk of outcomes.

## 1. Introduction

The Institute for Health Metrics and Evaluation showed that cases of lower respiratory tract infections worldwide increased from 414.3 million to 488.9 million between 1990 and 2019s (The Institute for Health Metrics and Evaluation (IHME), Global Health data exchange, GBD results tool, 2019). Due to the potential impact of accumulated hyperglycemia and oxidative stress, persons with diabetes showed reduced lung function and impaired neutrophil capability (Kornum et al., 2008; Gan, 2013). Studies have shown that persons with diabetes have a 1.2-to 2.6-fold higher risk of pneumonia than those without diabetes (Kornum et al., 2008; Harding et al., 2020). Recently, the incidence of macrovascular and microvascular complications in persons with type 2 diabetes (T2D) has decreased in many countries, possibly attributable to the aggressive control of blood pressure, lipids, and glucose levels. However, the occurrence of pneumonia is still on the rise (Wang et al., 2019; Pearson-Stuttard et al., 2022). The Taiwan Diabetes Atlas reported that the risks of hospitalization and mortality from pneumonia significantly increased from 2005 to 2014 in persons with T2D (Li et al., 2019; Wang et al., 2019). However, the diabetes guidelines for pneumonia management are limited (American Diabetes Association, 2021).

Peroxisome proliferator-activated receptors (PPARs) belong to a large superfamily of nuclear hormone receptors for retinoid, glucocorticoid, and thyroid hormones. PPARs are ligand-activated transcription factors crucial for regulating glucose homeostasis, adipocyte proliferation, atherosclerosis, cell cycle control, and inflammation (Zingarelli and Cook, 2005). Thiazolidinediones (TZDs) are the synthetic ligands of PPARs. Studies have demonstrated that in addition to improving insulin resistance, TZDs have anti-inflammatory and immunomodulatory properties. Preclinical studies have shown that TZDs can decrease neutrophil recruitment, downregulate inflammatory cytokines, and attenuate inflammation in acute lung injury (Zingarelli and Cook, 2005; Grommes et al., 2012). Thus, TZDs can influence the development or progression of pneumonia. One meta-analysis of 13 randomized clinical trials revealed that TZDs could moderately increase the risk of pneumonia in patients with T2D (Singh et al., 2011). However, pneumonias were adverse events of these trials, and most trials had low event rates. Without individual patient data, the pooled results may be worrying. Therefore, we conducted this nationwide cohort study to compare the risk of pneumonia between TZD users and nonusers to assess the impact of TZDs on pneumonia development or progression in persons with T2D.

## 2. Materials and methods

### 2.1. Study population

The Bureau of National Health Insurance implemented Taiwan’s National Health Insurance (NHI) program in 1995. The NHI program is a compulsory insurance system. The government and customers pay most of the premium, and the public only pays a small percentage. Approximately 99% of Taiwan’s 23 million persons joined the NHI program in 2000 (Cheng, 2003). All personal information of the insured, including sex, age, area of residence, insurance premium, diagnoses, medical procedures, and prescriptions, are recorded in the NHI Research Database (NHIRD). The diagnosis was based on the International Classification of Diseases, Ninth and Tenth Revision, Clinical Modification (ICD-9/10-CM). The NHIRD linked to the National Death Registry to verify mortality information. This study was approved by the Research Ethics Committee of China Medical University and Hospital [CMUH110-REC1-038 (CR-1)]. The identifiable information of the participants and caregivers was scrambled and encrypted before release to protect individual privacy. Informed consent was waived by the Research Ethics Committee.

### 2.2. Study design

We identified participants who were newly diagnosed with T2D between January 1, 2000, and December 31, 2017, and followed them until December 31, 2018. The diagnosis of T2D was based on ICD codings (ICD-9-CM codes: 250, except 250.1x; ICD-10-CM: E11) for at least 2 outpatient visits or one hospitalization. The algorithm for using ICD codes to define T2D was validated by a study in Taiwan with an accuracy of 74.6% (Lin et al., 2005). Participants were excluded (Supplementary Figure 1) under the following conditions: (The Institute for Health Metrics and Evaluation (IHME), Global Health data exchange, GBD results tool, 2019) age, below 20 or above 80 years; (Gan, 2013) missing age or sex information; (Kornum et al., 2008) diagnosis of type 1 diabetes (Supplementary Table 1), heart failure, or hepatic failure; (Harding et al., 2020) diagnosis of T2D established before January 1, 2000, to exclude prevalent cases.

### 2.3. Procedures

We defined the first date of TZD use as the index date. Participants who never received TZD treatment served as controls. We recorded the same period from the diagnosis of T2D to the use of TZDs as the index date for the control cases. Some related variables, checked and matched between TZD users and nonusers, were as follows: age (20–40, 41–60, 60–79 years), sex, obesity, smoking status; comorbidities, including alcohol-related disorders, hypertension, dyslipidemia, coronary artery disease (CAD), stroke, peripheral arterial occlusive disease (PAOD), chronic kidney disease (CKD), pneumonia, chronic obstructive pulmonary disease (COPD), liver cirrhosis, psychosis, depression, diagnosed within 1 year before the index date; medications, including oral antidiabetic drugs (OAD), insulin, statin, aspirin, corticosteroid, and immunosuppressants, used during the follow-up period. We calculated the Charlson Comorbidity Index (CCI), Diabetes Complication Severity Index (DCSI) score (Meduru et al., 2007; Young et al., 2008), and the number of oral antidiabetic drugs to evaluate the severity of T2D.

### 2.4. Main outcomes

The observed main outcomes of this study were hospitalization for all-cause pneumonia, hospitalization for bacterial pneumonia, invasive mechanical ventilation (IMV) use, and death due to pneumonia. One study in Taiwan validated the algorithm of using ICD codes to define pneumonia, with a sensitivity of 92.3–94.7% (Su et al., 2014). We calculated the events, person-years, and incidence rates for these outcomes during the follow-up period. We compared the cumulative incidences of the main outcomes between TZD users and nonusers.

### 2.5. Statistical analysis

We used propensity-score matching to optimize the relevant covariates between TZD users and nonusers (D’Agostino, 1998). The propensity score for each participant was estimated using non-parsimonious multivariable logistic regression, with TZD use as the dependent variable. We included 35 clinically related covariates as independent variables (Table 1). The nearest-neighbor algorithm was adopted to construct matched pairs, assuming the standardized mean difference (SMD) value <0.1 to be a negligible difference between the study and comparison cohorts.

**Table 1:** Baseline characteristics of participants with T2D with and without TZD use.

Variables	Before PSM					After PSM				
	Non-TZD		TZD		*p*-value	Non-TZD		TZD		SMD
	(*N* = 159,103)		(*N* = 52,147)		(*N* = 46,763)		(*N* = 46,763)	
	*n*	%	*n*	%	n	%	*n*	%
Sex					0.066					0.018
Female	76,944	48.36	24,977	47.90		21,458	45.89	21,877	46.78	
Male	82,159	51.64	27,170	52.10		25,305	54.11	24,886	53.22	
Age					<0.001					
20–40	13,712	8.62	3,297	6.32		2,982	6.38	2,980	6.37	<0.001
41–60	70,402	44.25	25,677	49.24		22,407	47.92	22,391	47.88	0.001
60–79	74,989	47.13	23,173	44.44		21,374	45.71	21,392	45.75	0.001
Mean, (SD)^a^	58.75	(12.4)	58.54	(11.33)	0.0003	58.8	(11.47)	58.81	(11.45)	<0.001
Obesity	5,043	3.17	1,551	2.97	0.026	1,333	2.85	1,368	2.93	0.004
Smoking status	4,322	2.72	1,133	2.17	<0.001	983	2.10	1,074	2.30	0.013
Comorbidities										
Alcohol-related disorders	5,711	3.59	1,674	3.21	<0.001	1,443	3.09	1,556	3.33	0.014
Hypertension	99,983	62.84	36,511	70.02	<0.001	32,518	69.54	32,527	69.56	0.000
Dyslipidemia	102,868	64.66	36,847	70.66	<0.001	32,197	68.85	32,589	69.69	0.018
Coronary artery disease	42,226	26.54	15,247	29.24	<0.001	13,194	28.21	13,323	28.49	0.006
Stroke	25,722	16.17	9,101	17.45	<0.001	7,921	16.94	8,170	17.47	0.014
PAOD	7,208	4.53	2,587	4.96	<0.001	2081	4.45	2,268	4.85	0.019
Chronic kidney disease	24,415	15.35	8,598	16.49	<0.001	7,151	15.29	7,515	16.07	0.021
Pneumonia	11,050	6.95	2,891	5.54	<0.001	2,860	6.12	2,572	5.50	0.026
COPD	28,464	17.89	8,322	15.96	<0.001	7,125	15.24	7,473	15.98	0.021
Liver cirrhosis	11,620	7.30	3,705	7.10	0.129	3,194	6.83	3,394	7.26	0.017
Psychosis	15,496	9.74	3,947	7.57	<0.001	3,466	7.41	3,658	7.82	0.015
Depression	10,012	6.29	2,449	4.70	<0.001	2,143	4.58	2,293	4.90	0.015
CCI					<0.001					
0	106,918	67.20	30,950	59.35		28,447	60.83	27,970	59.81	0.021
1	22,496	14.14	9,778	18.75		8,356	17.87	8,457	18.08	0.006
≥2	29,689	18.66	11,419	21.90		9,960	21.30	10,336	22.10	0.020
DCSI					<0.001					
0	53,217	33.45	12,882	24.70		12,738	27.24	12,161	26.01	0.028
1	32,721	20.57	10,181	19.52		9,187	19.65	9,221	19.72	0.002
≥2	73,165	45.99	29,084	55.77		24,838	53.11	25,381	54.28	0.023
Medication										
Metformin	80,512	50.60	46,852	89.85	<0.001	41,426	88.59	41,468	88.68	0.003
Sulfonylurea	65,753	41.33	46,520	89.21	<0.001	41,136	87.97	41,136	87.97	<0.001
DPP-4 inhibitor	13,848	8.70	8,191	15.71	<0.001	7,311	15.63	7,305	15.62	<0.001
AGI	12,992	8.17	15,344	29.42	<0.001	10,061	21.51	10,097	21.59	0.002
SGLT2 inhibitors	473	0.30	311	0.60	<0.001	275	0.59	275	0.59	<0.001
OAD numbers					<0.001					
0–1	98,921	62.17	6,790	13.02		7,081	15.14	6,790	14.52	0.018
2–3	56,985	35.82	41,848	80.25		36,634	78.34	37,354	79.88	0.038
>3	3,197	2.01	3,509	6.73		3,048	6.52	2,619	5.60	0.038
Insulin	66,868	42.03	25,883	49.63	<0.001	22,240	47.56	22,674	48.49	0.019
Statin	66,777	41.97	27,411	52.56	<0.001	23,951	51.22	24,196	51.74	0.010
Aspirin	73,517	46.21	28,309	54.29	<0.001	24,474	52.34	24,889	53.22	0.018
Corticosteroid	36,234	22.77	10,480	20.10	<0.001	9,632	20.60	9,735	20.82	0.005
Immunosuppressants	474	0.30	109	0.21	<0.001	111	0.24	105	0.22	0.003

SMD, standardized mean difference, SD, standard deviation, PAOD, peripheral arterial occlusive disease, COPD, chronic obstructive pulmonary disease, CCI, Charlson Comorbidity Index, DCSI, Diabetes Complication Severity Index, TZD, thiazolidinedione, DPP-4, dipeptidyl peptidase-4, AGI, alpha-glucosidase inhibitor, SGLT2, sodium-glucose cotransporter 2, OAD, oral antidiabetic drugs.

Data shown as *n* (%) or mean ± SD.

a Student’s *t*-test. The SMD value < 0.1 indicates a negligible difference between the study and control groups.

We used crude and multivariable-adjusted Cox proportional hazards models to compare outcomes between TZD users and nonusers. The results were presented as hazard ratios (HRs) and 95% confidence intervals (CIs) for TZD users compared with nonusers. This study is based on the intention-to-treat hypothesis. To calculate the observed risks, we censored the participants until the date of respective outcomes, death, or at the end of follow-up on December 31, 2018, whichever came first. The Kaplan–Meier method and log-rank tests were used to compare the cumulative incidences of hospitalization for all-cause pneumonia, bacterial pneumonia, IMV, and death due to pneumonia during the follow-up time between TZD users and nonusers. We compared the risk of hospitalization for all-cause pneumonia among different subgroups of age, sex, comorbidities, medications (rosiglitazone, pioglitazone, and others) for clinical applicability of results. We also assessed the cumulative duration (<153, 153–549, ≧550 days) and dose (<2,940, 2,940–10,009, ≧10,110 mg) of pioglitazone for the risks of hospitalization for all-cause pneumonia, bacterial pneumonia, IMV, and death due to pneumonia compared with no-use of TZDs to explore the dose relationship. We performed a stratified analysis to see the effect of TZD vs. non-TZD in the risk of all-cause pneumonia stratified by the subgroups of metformin use vs. no-use, SU use vs. no-use, DPP-4 inhibitor use-vs. no-use trying to determine whether other hypoglycemic agents have effect on pneumonia risk; stratified by patient’s resident areas of the Northern, Central, Southern, and Eastern Taiwan trying to see whether the different environmental exposures have different effect on pneumonia risk.

A two-tailed value of *p* <0.05 was considered significant. SAS (version 9.4; SAS Institute, Cary, NC, United States) was used for statistical analysis.

## 3. Results

### 3.1. Participants

From January 1, 2000, to December 31, 2017, we identified 338,361 participants with newly diagnosed T2D. Of these, 52,147 were TZD users, and 159,103 were nonusers (Supplementary Figure 1). After excluding unsuitable participants, 1: 1 propensity-score matching was used to construct 46,763 pairs of TZD users and nonusers. In the matched cohorts (Table 1), 46.34% of the participants were female; the mean (SD) age was 58.81 (11.46) years. The mean follow-up time for TZD users and nonusers was 7.80 (4.65) years and 5.21 (3.79) years, respectively.

### 3.2. Main outcomes

In the matched cohorts (Table 2), 7,753 (16.57%) TZD users and 5,220 (11.16%) nonusers were hospitalized for all-cause pneumonia during the follow-up time (incidence rate: 21.79 vs. 21.82 per 1,000 person-years). In the multivariable model, TZD users showed a significantly lower risk of hospitalization for all-cause pneumonia than nonusers (aHR = 0.92, 95% CI = 0.88–0.95). Compared with nonusers, TZD users also showed significantly lower risks of hospitalization for bacterial pneumonia (aHR 0.95, 95%CI 0.91–0.99), IMV (aHR 0.80, 95% CI 0.77–0.83), and death due to pneumonia (aHR 0.73, 95% CI 0.64–0.82).

**Table 2:** Incidence rate and hazard ratio of main outcomes between TZD use and no-use in patients with T2D.

**Outcomes**	**Non-TZD**			**TZD**			**cHR**	**(95% CI)**	***p*-value**	**aHR**^**a**^	**(95% CI)**	***p*-value**
	**N**	**PY**	**IR**	**N**	**PY**	**IR**						
Hospitalization for all-cause pneumonia		239,195	21.82	7,753	355,791	21.79	0.93	(0.90, 0.97)	<0.001	0.92	(0.88, 0.95)	<0.001
Hospitalization for bacterial pneumonia	3,738	241,514	15.48	5,448	360,389	15.12	0.96	(0.92, 1.00)	0.065	0.95	(0.91, 0.99)	0.014
Invasive mechanical ventilation	4,405	244,067	18.05	5,468	367,239	14.89	0.81	(0.78, 0.85)	<0.001	0.80	(0.77, 0.83)	<0.001
Death due to pneumonia	469	248,600	1.89	638	376,022	1.70	0.74	(0.65, 0.83)	<0.001	0.73	(0.64, 0.82)	<0.001

PY, person-years, IR, incidence rate, per 1,000 person-years, cHR, crude hazard ratio, aHR, adjusted hazard ratio.

aHR^a^, multivariable analysis adjusted for sex, age, comorbidities, CCI, DCSI scores, insulin, statin, aspirin, item, and number of oral antidiabetic drugs.

The Kaplan–Meier analysis showed that the cumulative incidences of hospitalization for all-cause pneumonia, IMV use, and death due to pneumonia were significantly lower in TZD users than nonusers (Log-rank test value of *p*<0.001). However, the cumulative incidence of hospitalization for bacterial pneumonia was non-significantly lower in TZD users than in nonusers (Log-rank test value of *p* = 0.066) (Supplementary Figure 2).

### 3.3. Subgroup analysis

We assessed the variables associated with the risk of hospitalization for all-cause pneumonia and found a significantly lower risk among participants using pioglitazone and statin. However, males, older age, participants with alcohol-related disorders, chronic kidney disease, COPD, depression, higher numbers of oral antidiabetic drugs, insulin, and aspirin use had a significantly higher risk of hospitalization for all-cause pneumonia (Table 3).

**Table 3:** Risk of hospitalization for all-cause pneumonia in patients with T2D stratified by variables.

**Variables**	***n***	**PY**	**IR**	**cHR**	**95% CI**	***p*-value**	**aHR**^**a**^	**95% CI**	***p*-value**
TZD nonusers	5,220	239,195	21.82	1.00	(Reference)	–	1.00	(Reference)	–
Rosiglitazone users	4,254	170,791	24.91	1.02	(0.97, 1.06)	0.4463	1.01	(0.96, 1.05)	0.7614
Pioglitazone users	3,499	185,000	18.91	0.86	(0.82, 0.9)	<0.001	0.85	(0.82, 0.89)	<0.001
Sex	.	.	.			–			
Female	5,591	289,164	19.34	1.00	(Reference)	–	1.00	(Reference)	–
Male	7,382	305,823	24.14	1.27	(1.22, 1.31)	<0.001	1.50	(1.45, 1.55)	<0.001
Age	.	.	.			–			
20–40	322	40,716	7.91	1.00	(Reference)	–	1.00	(Reference)	–
41–60	3,830	307,283	12.46	1.58	(1.41, 1.77)	<0.001	1.49	(1.33, 1.67)	<0.001
60–79	8,821	246,988	35.71	4.70	(4.2, 5.25)	<0.001	3.98	(3.55, 4.46)	<0.001
Mean, (SD)				1.07	(1.06,1.07)	<0.001	1.06	(1.05,1.06)	<0.001
Comorbidities									
Alcohol-related disorders	427	14,985	28.50	1.38	(1.25, 1.52)	<0.001	1.32	(1.19, 1.45)	<0.001
Chronic kidney disease	2,891	78,044	37.04	1.97	(1.89, 2.05)	<0.001	1.50	(1.43, 1.56)	<0.001
COPD	3,062	86,663	35.33	1.85	(1.77, 1.92)	<0.001	1.33	(1.27, 1.38)	<0.001
Depression	825	22,424	36.79	1.81	(1.68, 1.94)	<0.001	1.54	(1.43, 1.65)	<0.001
Medication									
OAD numbers									
0–1	1,590	98,589	16.13	1.00	(Reference)	–	1.00	(Reference)	–
2–3	10,976	477,738	22.97	1.46	(1.38, 1.54)	<0.001	1.30	(1.24, 1.38)	<0.001
>3	407	18,660	21.81	1.58	(1.41, 1.76)	<0.001	1.23	(1.10, 1.37)	<0.001
Insulin	7,432	261,655	28.40	1.76	(1.70, 1.82)	<0.001	1.49	(1.44, 1.55)	<0.001
Statin	5,574	274,591	20.30	0.92	(0.89, 0.95)	<0.001	0.77	(0.75, 0.80)	<0.001
Aspirin	8,482	297,047	28.55	1.95	(1.88, 2.03)	<0.001	1.36	(1.31, 1.41)	<0.001

PY, person-years, IR, incidence rate, per 1,000 person-years, cHR, crude hazard ratio, aHR, adjusted hazard ratio, COPD, chronic obstructive pulmonary disease, OAD, oral antidiabetic drugs.

aHR^a^, multivariable analysis adjusted for sex, age, comorbidities, CCI, DCSI scores, insulin, statin, aspirin, item, and number of oral antidiabetic drugs.

### 3.4. Cumulative duration and dose of pioglitazone

We investigated the association between the cumulative duration of pioglitazone use and the risks of hospitalization for all-cause pneumonia, bacterial pneumonia, IMV, and death due to pneumonia (Table 4). A longer cumulative duration of pioglitazone use was associated with further lower risks of hospitalization for all-cause pneumonia, bacterial pneumonia, IMV, and death due to pneumonia compared with no-use of TZDs. The value of ps for the trend were all significant (Table 4).

**Table 4:** Hazard ratios and 95% confidence intervals for outcomes with the cumulative duration of pioglitazone use.

**Variables**	**Event**	**PY**	**IR**	**cHR**	**(95% CI)**	***p* value**	**aHR**^**a**^	**(95% CI)**	***p*-value**
Hospitalization for all-cause pneumonia					
No-use of TZDs	5,220	239,195	21.82	1	(Reference)	–	1	(Reference)	–
Cumulative days of pioglitazone use (days)
<153	1,417	53,467	26.50	1.22	(1.15, 1.3)	<0.001	1.18	(1.11, 1.25)	<0.001
153–549	1,228	60,978	20.14	0.93	(0.87, 0.99)	0.02	0.96	(0.90, 1.02)	0.19
≧550	854	70,555	12.10	0.54	(0.5, 0.58)	<0.001	0.56	(0.52, 0.60)	<0.001
P for trend									<0.001
Hospitalization for bacterial pneumonia					
no-use of TZDs	3,738	241,514	15.48	1	(Reference)	–	1	(Reference)	–
Cumulative days of pioglitazone use (days)
<153	913	54,273	16.82	1.09	(1.01, 1.17)	0.027	1.09	(1.02, 1.17)	0.02
153–549	766	61,815	12.40	0.8	(0.74, 0.86)	<0.001	0.88	(0.81, 0.95)	0.001
≧550	502	71,142	7.06	0.45	(0.41, 0.5)	<0.001	0.49	(0.44, 0.54)	<0.001
P for trend									<0.001
Invasive mechanical ventilation					
No-use of TZDs	4,405	244,067	18.05	1	(Reference)	–	1	(Reference)	–
Cumulative days of pioglitazone use (days)
<153	987	55,155	17.90	0.99	(0.93, 1.07)	0.88	1.01	(0.93, 1.07)	0.98
153–549	749	62,609	11.96	0.67	(0.62, 0.72)	<0.001	0.71	(0.66, 0.77)	<0.001
≧550	463	71,979	6.43	0.36	(0.32, 0.39)	<0.001	0.38	(0.35, 0.42)	<0.001
P for trend									<0.001
Death due to pneumonia					
No-use of TZDs	469	248,600	1.89	1	(Reference)	–	1	(Reference)	–
Cumulative days of pioglitazone use (days)
<153	113	56,537	2.00	1.09	(0.89, 1.34)	0.41	1.06	(0.86, 1.31)	0.57
153–550	90	63,878	1.41	0.76	(0.61, 0.96)	0.02	0.83	(0.66, 1.04)	0.11
>550	59	72,706	0.81	0.4	(0.3, 0.52)	<0.001	0.44	(0.33, 0.57)	<0.001
P for trend									<0.001

PY, person-years, IR, incidence rate, per 1,000 person-years, cHR, crude hazard ratio, aHR, adjusted hazard ratio.

aHR^a^, multivariable analysis adjusted for sex, age, comorbidities, CCI, DCSI scores, insulin, statin, aspirin, item, and number of oral antidiabetic drugs.

We also observed an association between the cumulative dose of pioglitazone and the risks of hospitalization for all-cause pneumonia, bacterial pneumonia, IMV, and death due to pneumonia (Table 4). The higher cumulative dose of pioglitazone use was associated with further lower risks of hospitalization for all-cause pneumonia, bacterial pneumonia, IMV, and death due to pneumonia compared with no-use of TZDs; the value of ps for the trend were all significant (Table 5).

**Table 5:** Hazard ratios and 95% confidence intervals for outcomes with the cumulative dose of pioglitazone use.

**Variables**	**Event**	**PY**	**IR**	**cHR**	**(95% CI)**	***p* value**	**aHR**^**a**^	**(95% CI)**	***p*-value**
Hospitalization for all-cause pneumonia									
No-use of TZDs	5,220	239,195	21.82	1	(Reference)	–	1	(Reference)	–
Cumulative dose of pioglitazone (mg)	
<2,940	1,627	50,547	32.19	1.49	(1.41, 1.58)	<0.001	1.39	(1.31, 1.47)	<0.001
2,940–10,009	1,091	54,383	20.06	0.93	(0.87, 0.99)	0.024	0.94	(0.88, 1)	0.07
≧10,110	781	80,071	9.75	0.43	(0.4, 0.47)	<0.001	0.46	(0.43, 0.5)	<0.001
P for trend									<0.001
Hospitalization for bacterial pneumonia					
No-use of tzds	3,738	241,514	15.48	1	(Reference)	–	1	(Reference)	–
Cumulative dose of pioglitazone (mg)	
<2,940	1,057	51,329	20.59	1.33	(1.24, 1.42)	<0.001	1.3	(1.21, 1.39)	<0.001
2,940–10,009	671	55,094	12.18	0.79	(0.72, 0.85)	<0.001	0.85	(0.78, 0.92)	<0.001
≧10,110	453	80,808	5.61	0.36	(0.32, 0.39)	<0.001	0.4	(0.36, 0.44)	<0.001
P for trend									<0.001
Invasive mechanical ventilation									
No-use of TZDs	4,405	244,067	18.05	1	(Reference)	–	1	(Reference)	–
Cumulative dose of pioglitazone (mg)	
<2,940	1,050	52,109	20.15	1.12	(1.05, 1.2)	<0.001	1.1	(1.02, 1.17)	0.009
2,940–10,009	654	55,845	11.71	0.65	(0.6, 0.71)	<0.001	0.69	(0.64, 0.75)	<0.001
≧10,110	495	81,789	6.05	0.34	(0.31, 0.37)	<0.001	0.37	(0.34, 0.4)	<0.001
P for trend									<0.001
Death due to pneumonia					
No-use of TZDs	469	248,600	1.89	1	(Reference)	–	1	(Reference)	–
Cumulative dose of pioglitazone (mg)	
<2,940	114	53,431	2.13	1.18	(0.96, 1.44)	0.12	1.1	(0.89, 1.35)	0.37
2,940–10,009	93	56,936	1.63	0.89	(0.71, 1.11)	0.30	0.94	(0.75, 1.17)	0.58
≧10,110	55	82,752	0.66	0.33	(0.25, 0.43)	<0.001	0.38	(0.28, 0.5)	<0.001
P for trend									<0.001

PY, person-years, IR, incidence rate, per 1,000 person-years, cHR, crude hazard ratio, aHR, adjusted hazard ratio.

aHR^a^, multivariable analysis adjusted for sex, age, comorbidities, CCI, DCSI scores, insulin, statin, aspirin, item, and number of oral antidiabetic drugs.

### 3.5. Additional analyses

The stratified analysis of different 4 regions of Taiwan on all-cause pneumonia risk between TZD users vs. nonusers seems to be consistent (Supplementary Table 2). But among other hypoglycemic agents, combined use of DPP-4 inhibitor and TZD seems to have a higher risk of hospitalization for all-cause pneumonia (Supplementary Table 2).

## 4. Discussion

This study demonstrated that TZD use was associated with significantly lower risks of hospitalization for pneumonia, IMV, and death due to pneumonia than TZD no-use in persons with T2D. The subgroup analysis revealed that the reduced risk of pneumonia by TZDs could be due to pioglitazone use. The dose–response analysis showed that longer cumulative duration and a higher cumulative dose of pioglitazone were associated with further lower risks of these outcomes.

Studies have shown that patients with diabetes have a higher risk of pneumonia than those without diabetes (Kornum et al., 2008; Harding et al., 2020). Patients with suboptimal glycemic control showed a higher risk of pneumonia (Kornum et al., 2008). Our study also revealed that older people, persons with comorbidities, using more oral antidiabetic drugs, insulin, and aspirin had a higher risk of pneumonia. However, patients using TZDs, especially pioglitazone and statin, had a lower risk of hospitalization for all-cause pneumonia. Reports show that statin use is associated with a lower risk of pneumonia due to its potential anti-inflammatory effect (Macedo et al., 2014). However, a systemic review and meta-analysis by Sigh et al. revealed that TZD use was associated with a modestly elevated risk of pneumonia [relative risk (RR) 1.4(1.08–1.82)] (Singh et al., 2011). Gorricho et al. conducted a nested case–control study comparing the use of oral antidiabetic drugs and the risk of community-acquired pneumonia. They showed that TZDs combined with other antidiabetic drugs were associated with an increased risk of pneumonia compared to metformin plus sulfonylureas (Gorricho et al., 2017). The different results obtained from the three studies could be due to differences in the methodology and the study population. Moreover, Shih et al. conducted a case–control study and showed that TZD use was associated with a modest reduction of sepsis risk compared to TZD no-use in persons with T2D (Shih et al., 2015). To our knowledge, our research is the first study designed to compare the risk of pneumonia between TZD users and nonusers and suggest that TZD use may attenuate the risk of hospitalization for all-cause [aHR 0.92 (0.88, 0.95)] and bacterial pneumonia [aHR 0.95 (0.91, 0.99)]. This result may not be affected by the different resident environment of Taiwan. Bu if the patient is combined use of DPP-4 inhibitor and TZD seems to make the TZD lose their protective effect against all-cause pneumonia for reasons that are unclear. This study also showed that pioglitazone, not rosiglitazone, could reduce the risk of pneumonia. Rosiglitazone is a PPARγ agonist, but pioglitazone has both α and γ effects. Each TZD has different patterns of effects on the regulation of gene transcription (Kung and Henry, 2012). Previous studies have shown that the impact of pioglitazone and rosiglitazone on cardiovascular diseases was different (Kung and Henry, 2012). Preclinical studies have also found that the effect of pioglitazone and rosiglitazone on inflammation may be dissimilar (Zingarelli and Cook, 2005; Singh et al., 2011). More research is needed to determine any difference in the effectiveness of pioglitazone and rosiglitazone in the risk of pneumonia.

Diabetes may reduce lung function and pulmonary diffusion capacity due to microangiopathic changes in the lungs (Pitocco et al., 2012). Animal studies have demonstrated that pioglitazone can attenuate endotoxin-induced acute lung injury and pulmonary edema (Grommes et al., 2012). Kim et al. have shown that insulin sensitizers (metformin or TZDs) were independently associated with improvements in forced vital capacity (FVC) in persons with T2D and COPD (Kim et al., 2010). Our study demonstrated that TZDs were significantly associated with a lower risk of invasive mechanical ventilation than non-TZDs in persons with T2D [aHR 0.80 (0.77, 0.83)]. More studies are needed to explore the effect of TZDs on respiratory function, pulmonary microangiopathy, and inflammation.

Although the availability of excellent antibiotics has resulted in a significant reduction in mortality from pneumonia, the reduction in mortality within 7 days of the onset of pneumonia is not prominent. This finding may be due to the inability of antibiotics to rapidly reduce inflammatory events in the lungs (Corrales-Medina and Musher, 2011). Pneumonia may also be an important factor in accelerating premature death in persons with T2D and multimorbidity (Fine et al., 1996; Li et al., 2019; Pearson-Stuttard et al., 2022). Notably, this study showed that TZDs were significantly associated with a lower risk of death due to pneumonia [aHR 0.73 (0.64, 0.82)]. This finding may be attributable to the reduced risk of hospitalization for pneumonia and IMV support by TZD use. This study also showed that TZDs were more effective in protecting against hospitalization for all-cause pneumonia, IMV use, and death due to pneumonia than against hospitalization for bacterial pneumonia (Table 2), which may indicate that the anti-inflammatory effect of TZDs on protection against pneumonia may be greater than their antibacterial effect.

The possible grounds for TZDs to decrease the development and progression of pneumonia in persons with T2D are as follows: (The Institute for Health Metrics and Evaluation (IHME), Global Health data exchange, GBD results tool, 2019) pharmacological activation of PPARγ by TZDs can inhibit proinflammatory gene expression and reduce the production of C-reactive protein (CRP), tumor necrosis factor (TNF)-α, interleukin (IL)- 1β, IL-6, inducible nitric oxide synthase (iNOS), inducible cyclooxygenase (COX)-2, matrix metalloproteinase (MMP)-9, macrophage chemoattractant protein (MCP)-1, and plasminogen activator inhibitor (PAI)-1 (Zingarelli and Cook, 2005; Hanefeld et al., 2007; Gan, 2013). The induction of heat shock proteins by PPARγ ligands may alter the activation of nuclear factor-*k*B (NF-*k*B) and regulate inflammation (Zingarelli and Cook, 2005; Kornum et al., 2008). TZDs may augment CD36 expression, tether apoptotic cells to macrophages to promote efferocytosis, and evoke an anti-inflammatory response with the resolution of tissue injury (Zingarelli and Cook, 2005; Lea et al., 2014; Harding et al., 2020). Studies also showed that TZDs could increase IL-10 levels, enhance neutrophil recruitment to the infection foci, raise fibroblast growth factor (FGF) 21 levels, and improve survival in animals with sepsis (Trevelin et al., 2017; Pearson-Stuttard et al., 2022). PPARγ may play a role in the differentiation of naive T cells to effector T cells and improve adaptive immunity (Daynes and Jones, 2002; Wang et al., 2019). Preclinical studies demonstrated that TZDs could have direct antibacterial activity (Stegenga et al., 2009; Masadeh et al., 2011; Li et al., 2019). In animal models of lung injury, TZDs decreased pulmonary edema, fibrosis, inflammation, and mortality (Belvisi and Mitchell, 2009; Grommes et al., 2012).

This study has some limitations. First, this NHI dataset lacks complete information on dietary patterns, smoking habits, alcohol drinking, nutritional state, physical activity, vaccination status, and family history. It does not contain data on glucose, hemoglobin A1C, biochemical and microbiological tests, immune condition, pulmonary function tests, and imaging studies, which prevents a better understanding of the patient’s health status and the severity of diabetes. However, we matched the demographic information on sex and age to achieve a balance between the study and control groups. We also matched the items and number of oral antidiabetic drugs, insulin use, and DCSI scores to balance the severity of diabetes and increase the comparability between the study and comparison groups. Second, we had information on prescriptions, but patient compliance with medications was unknown. We could not obtain clues to the doctor’s preference for prescribing and the patient’s choice of medications from this database. Third, almost all the participants in this study were Chinese, and hence, the results may not be generalizable to other races. Fourth, cohort studies are usually associated with few unknown or unobserved confounding factors; therefore, randomized controlled trials are needed to verify our results. Finally, because TZDs have the concern of heart failure risk, if we want to use TZDs to reduce the risk of hospitalization for pneumonia, we must pay close attention to patients for signs and symptoms of heart failure to avoid unexpected harm to patients.

Persons with diabetes are more likely to contract and die from pneumonia than those without diabetes. Although the incidence of vascular complications has decreased, the occurrence of pneumonia is still rising. In addition to recommending persons with diabetes to receive influenza and pneumococcal vaccinations, perhaps TZD use may be an option to attenuate the morbidity and mortality of pneumonia. Additional studies are warranted to clarify all the effects of TZDs potentially linked to pneumonia.

## Data availability statement

The data analyzed in this study is subject to the following licenses/restrictions: Data of this study are available from the National Health Insurance Research Database (NHIRD) published by Taiwan National Health Insurance (NHI) Administration. The data utilized in this study cannot be made available in the paper, the supplemental files, or in a public repository due to the “Personal Information Protection Act” executed by Taiwan government starting from 2012. Requests for data can be sent as a formal proposal to the NHIRD Office (https://dep.mohw.gov.tw/DOS/cp-2516-3591-113.html) or by email to stsung@mohw.gov.tw. Requests to access these datasets should be directed to stsung@mohw.gov.tw

## Ethics statement

The studies involving human participants were reviewed and approved by Research Ethics Committee of China Medical University and Hospital. The ethics committee waived the requirement of written informed consent for participation.

## Author contributions

F-SY: study concept, design, and drafting the manuscript, critical revision of the manuscript for important intellectual content, and study supervision. JW: critical revision of the manuscript for important intellectual content, technical and material support, and study supervision. Y-TH: data acquisition, analysis, interpretation, and statistical analysis. CH: data acquisition, interpretation, funding acquisition, technical or material support, and critical revision of the manuscript for important intellectual content. C-MH: study concept and design, data acquisition, analysis, interpretation, drafting the manuscript, critical revision of the manuscript for important intellectual content, statistical analysis, funding acquisition, technical or material support, and study supervision. C-CH: analysis and interpretation of the data, drafting the manuscript, critical revision of the manuscript for important intellectual content, and study supervision. All authors contributed to the article and approved the submitted version.

## Funding

This study is supported in part by the Ministry of Science and Technology (MOHW109-TDU-B-212-114004) and China Medical University Hospital (DMR-111-105). This work received grant support from Taipei Veterans General Hospital (V101C-156, V108C-172, V109C-189). These funding agencies had no role in the study design, data collection and analysis, decision to publish, or manuscript preparation. No organization provided funds to assist with the preparation of this paper, and data analysis was not performed by employees of funders or any author who received funding. The funders had no role in study design, data collection, data analysis, the decision to publish, or manuscript preparation. This study received no additional external funding.

## Conflict of interest

The authors declare that the research was conducted in the absence of any commercial or financial relationships that could be construed as a potential conflict of interest.

## Publisher’s note

All claims expressed in this article are solely those of the authors and do not necessarily represent those of their affiliated organizations, or those of the publisher, the editors and the reviewers. Any product that may be evaluated in this article, or claim that may be made by its manufacturer, is not guaranteed or endorsed by the publisher.
