# Supplementary material for: Thiazolidinediones lower the risk of pneumonia in patients with type 2 diabetes
Source: Front Microbiol. 2023 Feb 17;14:1118000. doi: 10.3389/fmicb.2023.1118000 (PMC9981669; doi:10.3389/fmicb.2023.1118000)
Supplement: Supplementary file 1 [file Table_1.DOCX]

Supplementary Material

Thiazolidinediones Lower the Risk of Pneumonia in Patients With Type 2 Diabetes

Fu-Shun Yen, James Cheng-Chung Wei, Yu-Tung Hung, Chung Y. Hsu, Chii-Min Hwu^*^, Chih-Cheng Hsu^*^

*** Correspondence:** Chii-Min Hwu: [chhwu@vghtpe.gov.tw](mailto:chhwu@vghtpe.gov.tw); Chih-Cheng Hsu: cch@nhri.edu.tw

# Supplementary Figures and Table

## Supplementary Figures


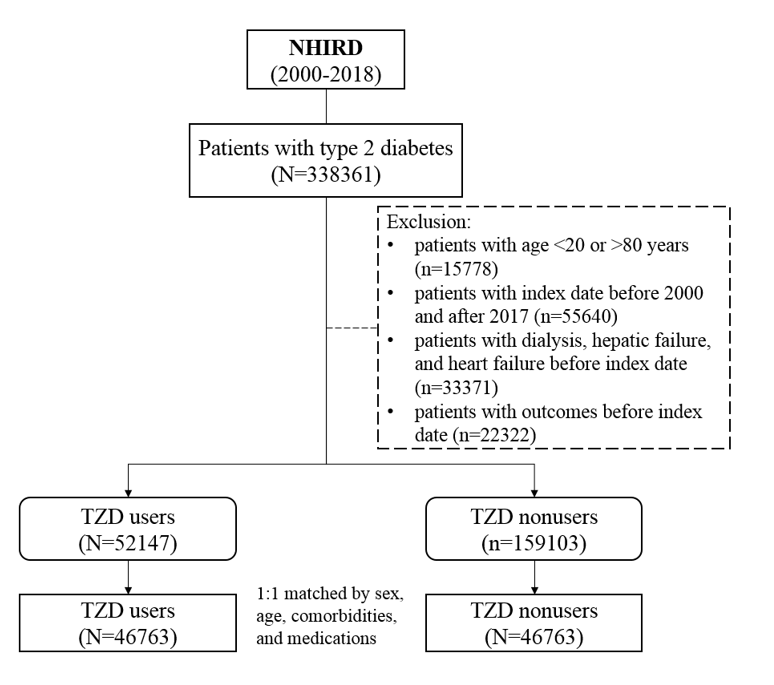


**SUPPLEMENTARY FIGURE 1 |** Flow chart of the identified process in this study.

**
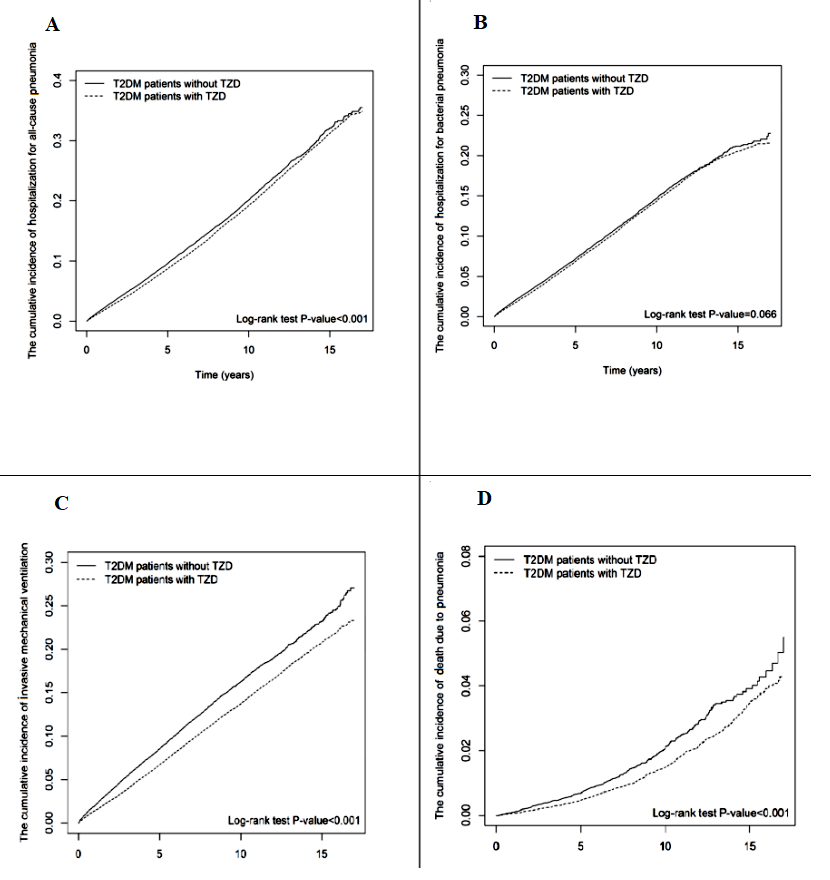
**

**SUPPLEMENTARY FIGURE 2 |** Cumulative incidences of **(A)** hospitalization for all-cause pneumonia, **(B)** hospitalization for bacterial pneumonia, **(C)** invasive mechanical ventilation, and **(D)** death due to pneumonia for TZDs users vs. TZDs nonusers.

## Supplementary Table

**SUPPLEMENTARY TABLE 1 |** Diseases and related ICD-9-CM, ICD-10-CM codes.

| **Disease** | **ICD-9-CM codes** | **ICD-10-CM codes** |
| --- | --- | --- |
| Type 2 diabetes | 250.xx, except 250.1x | E11 |
| Type 1 diabetes | 250.1x | E10 |
| Hepatic failure | 570, 572.2, 572.4, 572.8 | K72.00, K72.01, K72.10, K72.11, K72.90, K76.2, K72.90, K72.91, K76.7, K76.81 |
| Heart failure | 428 | I50 |
| Obesity | 278.02, 783.1, V85.2, 278.00, 649.1, V77.8, V85.3, 278.01, 649.2, V45.86, V85.4 | R63.5, E66.09, E66.1, E66.8, E66.9, Z13.89, E66.01, E66.2 |
| Smoking status | 305.1, 649.0, V15.82 | F17.200, F17.201, F17.210, F17.220, F17.221, F17.290, F17.291, Z87.891 |
| Alcohol-related disorders | 291, 303, 305.0, 571.0-571.3, V11.3, V79.1 | F10, K70.40, K70.41, K70.9 |
| Hypertension | 401–405, A26 | I10, I11, I12, I13, I15, N26.2 |
| Dyslipidemia | 272 | E71.30, E71.31, E71.32, E71.39, E75.21, E75.22, E75.23, E75.24, E75.25, E75.29, E75.3, E75.4, E75.5, E75.6, E77, E78.0, E78.1, E78.2, E78.3, E78.4, E78.5, E78.6, E78.70, E78.71, E78.72, E78.79, E78.8, E78.9 |
| Chronic kidney disease | 250.4x, 403.xx, 404.xx, 585.xx, 586.xx, 581.8x, 791.0x, 593 | E10.2, E10.65, E11.2, E11.65, E13.2, I12, I13, N03, N08, E10.21, E11.21, N05, N06, N07, N14, N15.0, N15.8, N15.9, N16, N17.1, N17.2, N18, N19 |
| Coronary artery disease | 410-414 | I20, I21, I22, I24, I25.1, I25.2, I25.3, I25.4, I25.5, I25.6, I25.7, I25.81, I25.82, I25.83, I25.84, I25.89, I25.9 |
| Stroke | 430-438 | G45.0, G45.1, G45.2, G45.3, G45.4, G45.8, G45.9, G46, I60, I61, I62, I63, I65, I66, I67.0, I67.1, I67.2, I67.3, I67.4, I67.5, I67.6, I67.7, I67.8, I67.9, I68, I69 |
| Peripheral arterial occlusive disease | 440.0, 440.20, 440.21, 440.22, 440.23, 440.24, 440.3, 440.4, 443.9, 443.81, 443.89 | I70.2, I70.92, I75.0, I73.9 |
| Chronic obstructive pulmonary disease | 491, 492, 496 | J41, J42, J44, J43, J44.9 |
| Liver cirrhosis | 571.5, 571.2, 571.6 | K70.2, K70.30, K70.31, K74.0, K74.1, K74.2, K74.60, K74.69, K74.3, K74.4, K74.5 |
| Psychosis | 290–299 | F20-29 |
| Depression | 311 | F32, F33 |
| All-cause pneumonia | 480-486 | J12-18 |
| Bacterial pneumonia | 481, 482.41, 482.8, 486 | J13-15 |
| Invasive mechanical ventilation | 96.7 | Z99.1 |

| **SUPPLEMENTARY TABLE 2 \|** Risk of hospitalization for all-cause pneumonia in type 2 diabetes patients relative to non-TZD and TZD stratified by variables. | | | | | | | | | | | | |
| --- | --- | --- | --- | --- | --- | --- | --- | --- | --- | --- | --- | --- |
|  | **Non-TZD** | | | **TZD** | | |  | | |  | | |
| **Variables** | **N** | **PY** | **IR** | **N** | **PY** | **IR** | **cHR** | **(95% CI)** | **p-value** | **aHR^a^** | **(95% CI)** | **p-value** |
| Area of Taiwan |  |  |  |  |  |  |  |  |  |  |  |  |
| Northern | 2144 | 113754 | 18.85 | 3145 | 163385 | 19.25 | 0.95 | (0.9, 1.00) | 0.072 | 0.95 | (0.90, 1.00) | 0.055 |
| Central | 1077 | 47647 | 22.60 | 1867 | 83523 | 22.35 | 0.90 | (0.83, 0.97) | 0.007 | 0.90 | (0.83, 0.97) | 0.005 |
| Southern | 1823 | 70092 | 26.01 | 2457 | 97928 | 25.09 | 0.92 | (0.86, 0.98) | 0.008 | 0.89 | (0.84, 0.95) | <0.001 |
| Eastern | 176 | 7701 | 22.85 | 284 | 10956 | 25.92 | 1.08 | (0.89, 1.3) | 0.451 | 0.94 | (0.78, 1.14) | 0.536 |
| Antidiabetic drugs |  |  |  |  |  |  |  |  |  |  |  |  |
| Metformin |  |  |  |  |  |  |  |  |  |  |  |  |
| No | 574 | 33815 | 16.97 | 845 | 47020 | 17.97 | 1.01 | (0.91, 1.13) | 0.792 | 0.98 | (0.88, 1.09) | 0.647 |
| Yes | 4646 | 205381 | 22.62 | 6908 | 308772 | 22.37 | 0.92 | (0.88, 0.95) | <0.001 | 0.91 | (0.87, 0.94) | <0.001 |
| Sulfonylurea |  |  |  |  |  |  |  |  |  |  |  |  |
| No | 343 | 29614 | 11.58 | 578 | 41696 | 13.86 | 1.12 | (0.98, 1.28) | 0.097 | 1.09 | (0.95, 1.24) | 0.239 |
| Yes | 4877 | 209581 | 23.27 | 7175 | 314096 | 22.84 | 0.92 | (0.88, 0.95) | <0.001 | 0.90 | (0.87, 0.94) | <0.001 |
| DPP-4 inhibitors |  |  |  |  |  |  |  |  |  |  |  |  |
| No | 4905 | 216636 | 22.64 | 7258 | 329092 | 22.05 | 0.91 | (0.88, 0.95) | <0.001 | 0.90 | (0.87, 0.93) | <0.001 |
| Yes | 315 | 22560 | 13.96 | 495 | 26700 | 18.54 | 1.29 | (1.12, 1.49) | <0.001 | 1.18 | (1.02, 1.36) | 0.027 |

Abbreviation: PY = person-years, IR = incidence rate, per 1000 person-years, cHR = crude hazard ratio, aHR = adjusted hazard ratio, TZD = thiazolidinedione, DPP-4 = dipeptidyl peptidase-4.

aHR^a^, multivariable analysis adjusted for sex, age, comorbidities, CCI, DCSI scores, insulin, statin, aspirin, item, and number of oral antidiabetic drug
